# Investigating variation in reported location of death: A comparison of administrative data sources

**DOI:** 10.23889/ijpds.v9i5.2811

**Published:** 2024-09-18

**Authors:** Tom Elliott, Barry Milne, Eileen Li, Andrew Sporle, Colin Simpson

**Affiliations:** 1 iNZight Analytics Ltd; 2 University of Auckland; 3 Victoria University of Wellington

How can we lower barriers to reuse of linked administrative data and improve quality at the same time? Linked data resources are expensive and complex, but accompanying readily-accessible metadata supports data reuse and quality control. However, metadata systems that are unsystematic or non-automated can lead to inconsistencies, making metadata use difficult even for experienced analysts.

Our national statistics office (NSO) maintains a research database (RD) of administrative datasets linkable at the individual level. With the NSO’s agreement, we extracted schema information (e.g. tables and variables) from the RD and obtained data dictionaries. R was used to extract information (e.g., descriptions, human-friendly names, codings) from the data dictionaries, which identified errors and inconsistencies that the NSO then fixed. A metadata database was collated using schema and data dictionary information about variables and datasets in the RD. Finally, a public web app was developed to enable exploration of the meta database by searching specific terms or navigating the hierarchical relationships.

We now work with the NSO data team to re-extract variables and add or update dictionaries ahead of the regular data updates. Metadata coverage is displayed on the app and used by our NSO to improve metadata quality and coverage. Our scripting workflow demonstrates the utility of automation in picking up errors and inconsistencies quickly, and before they can propagate by copy and paste. The web app, available to existing and new users, is now listed by the NSO as a “go-to” resource for using the linked administrative data resources.

